# Appropriateness of Allogeneic Red Blood Cell Transfusions in Non-Bleeding Patients in a Large Teaching Hospital: A Retrospective Study

**DOI:** 10.3390/jcm12041293

**Published:** 2023-02-06

**Authors:** Piotr F. Czempik, Dawid Wilczek, Jan Herzyk, Łukasz J. Krzych

**Affiliations:** 1 Department of Anaesthesiology and Intensive Care, Faculty of Medical Sciences in Katowice, Medical University of Silesia, 40-752 Katowice, Poland; 2 Transfusion Committee, University Clinical Center, Medical University of Silesia, 40-752 Katowice, Poland; 3 Students’ Scientific Society, Department of Anaesthesiology and Intensive Care, Faculty of Medical Sciences in Katowice, Medical University of Silesia, 40-752 Katowice, Poland

**Keywords:** anemia, hemoglobin, indication, patient blood management, red blood cell, transfusion, trigger

## Abstract

In hemodynamically stable patients, both anemia and red blood cell (RBC) transfusion may be detrimental to patients; hence, a decision regarding RBC transfusion should be based on thorough risk–benefit assessment. According to hematology and transfusion medicine organizations, RBC transfusion is indicated when recommended hemoglobin (Hb) triggers are met, and symptoms of anemia are present. The aim of our study was to examine the appropriateness of RBC transfusions in non-bleeding patients at our institution. We performed a retrospective analysis of all RBC transfusions performed between January 2022 and July 2022. The appropriateness of RBC transfusion was based on the most recent Association for the Advancement of Blood and Biotherapies (AABB) guidelines and some additional criteria. The overall incidence of RBC transfusions at our institution was 10.2 per 1000 patient-days. There were 216 (26.1%) RBC units appropriately transfused and 612 (73.9%) RBC units that were transfused with no clear indications. The incidence of appropriate and inappropriate RBC transfusions were 2.6 and 7.5 per 1000 patient-days, respectively. The most frequent clinical situations when RBC transfusion was classified as appropriate were: Hb < 70 g/L plus cognitive problems/headache/dizziness (10.1%), Hb < 60 g/L (5.4%), and Hb < 70 g/L plus dyspnea despite oxygen therapy (4.3%). The most frequent causes of inappropriate RBC transfusions were: no Hb determination pre-RBC transfusion (n = 317) and, among these, RBC transfused as a second unit in a single-transfusion episode (n = 260); absence of anemia sings/symptoms pre-transfusion (n = 179); and Hb concentration ≥80 g/L (n = 80). Although the incidence of RBC transfusions in non-bleeding inpatients in our study was generally low, the majority of RBC transfusions were performed outside recommended indications. Red blood cell transfusions were evaluated as inappropriate mainly due to multiple-unit transfusion episodes, absence of anemia signs and/or symptoms pre- transfusion, and liberal transfusion triggers. There is still the need to educate physicians on appropriate indications for RBC transfusion in non-bleeding patients.

## 1. Introduction

The decision regarding red blood cell (RBC) transfusion constitutes a daily challenge to hospital physicians [1]. Transfusion of RBC in massive bleeding saves lives. As an unequivocally benefit in the context of bleeding, RBC transfusion is supported by clinical practice guidelines [2]. RBC transfusion performed outside bleeding emergency has a more complicated risk–benefit profile. In hemodynamically stable patients, both anemia and RBC transfusion may be detrimental to patients; hence, a decision regarding RBC transfusion should be based on thorough risk–benefit assessment.

Anemia is an independent risk factor for myocardial ischemia, stroke, acute kidney injury, and infection [3]. Even mild anemia, defined as hemoglobin (Hb) concentration 110–130 g/L in men and 110–120 g/L in women, doubles long-term mortality in patients scheduled for major elective surgery or hospitalized in the intensive care unit (ICU) [4,5]. Anemia may lead to longer hospitalization [6]. Moreover, anemia may increase the odds of RBC transfusion [7].

On the other hand, transfusion of RBC may lead to prolonged respirator weaning or weaning failure [8], pneumonia [9] and other nosocomial infections, immunomodulation, transfusion-associated cardiac overload (TACO), transfusion-associated acute lung injury (TRALI), thromboembolic complications, allergic reactions, immunization, blood-born pathogen transmission, and early and delayed hemolytic reactions. Finally, RBC transfusion may lead to increased mortality [10] and longer hospitalization.

Taking these into account, decisions regarding RBC transfusion in non-emergency setting should follow thorough risk–benefit assessment in an individual patient. Transfusion of RBC was listed by the American Board of Internal Medicine Foundation’s Choosing Wisely Campaign as one of the top five procedures for which rationale should be questioned by physicians and patients [11]. According to various organizations, RBC should be transfused at a restrictive threshold (i.e., Hb < 70 g/L) [12]. Moreover, the decision regarding RBC transfusion should be based not solely on Hb concentration but also on symptoms of anemia [13] or broader clinical context [14]. Even elderly patients may, if properly managed, tolerate very low Hb concentrations [15]. At last, in deciding on RBC transfusion, some authors suggest using the so called physiologic RBC transfusion triggers [16,17,18].

The aim of our study was to examine the appropriateness of RBC transfusions in non-bleeding patients at our institution.

## 2. Materials and Methods

We performed a retrospective analysis of all RBC transfusions performed in non-bleeding patients between January 2022 and July 2022. We excluded RBCs that were transfused for bleeding (n = 301) or as preparation for surgery (n = 104). Our institution is a large tertiary medical center affiliated with a medical university with 644 hospital beds in two locations. Both subspecialty surgical and subspecialty medical disciplines are represented at the institution. Gastrointestinal surgery department admits patients scheduled for gastrointestinal oncologic and non-oncologic surgery. The oncologic surgery department is a small unit that admits patients scheduled for oncologic surgery of mostly the breast, thyroid, and pancreas. In the department of autoimmune and metabolic diseases, patients with various medical diagnoses are hospitalized; some of the patients are transferred from medical oncology departments due to deterioration after chemotherapy treatment. The clinical pharmacology department specializes in optimizing the pharmacotherapy of chronic conditions and treatment of patients with dyslipidemias. The intensive care unit (ICU) is a mixed medical–surgical department. There is no hematology department at the institution. Patients with hematological diseases are occasionally hospitalized in the departments of autoimmune and metabolic diseases and clinical pharmacology.

All data were retrieved from the hospital electronic health records (AMMS, Asseco Medical Solutions, Rzeszów, Poland). The percentage of patients who received at least a single RBC was calculated for the institution in general and per hospital department. Incidence of RBC transfusions was calculated as the number of transfusions per 1000 patient-days. Demographic and clinical data on RBC recipients were retrieved: age, sex, hospital department, presence of signs and/or symptoms of anemia pre- and post RBC transfusion, laboratory markers of anaerobic metabolism (central venous oxygen saturation—ScvO_2_, mixed venous oxygen saturation—SvO_2_, blood lactate) pre- and post RBC transfusion, and Hb concentration up to 24 h pre- and post RBC transfusion. Hemoglobin concentration was determined using a venous blood sample, which is considered a gold standard for Hb determination.

We evaluated appropriateness of RBC transfusion according to the most recent clinical practice guidelines from the Association for the Advancement of Blood and Biotherapies (AABB) [14]. When deciding on appropriateness of RBC transfusion, both Hb concentration and presence of signs and/or symptoms of anemia were taken into account. As an appropriate trigger for RBC transfusion, Hb concentration <70 g/L was used [12], whereas in patients with a history of coronary artery disease (CAD), Hb concentration < 80 g/L was used. All RBC transfusions with pre-transfusion Hb concentration < 60 g/L were classified as appropriate irrespective of anemia signs and/or symptoms. When pre-transfusion Hb concentration was 60–70 g/L, RBC transfusion was classified as appropriate when at least one sign and/or symptom of anemia was present, and/or at least one laboratory marker of anaerobic metabolism was abnormal. The signs and symptoms of anemia that were looked for in evaluating the appropriateness of RBC transfusion were: dizziness, lightheadedness, headache, problems with concentration, attention deficit, shortness of breath, tachycardia, hypotension, chest pain, and new electrocardiogram (ECG) changes. These cardiologic and respiratory signs and/or symptoms had to be present following attempts at improving patients’ tolerance of anemia through hemodynamic and oxygenation optimization. The most common markers of anaerobic metabolism at our institution, used as so-called physiologic transfusion triggers, were ScvO_2_ and lactate. The other markers that are used as physiologic transfusion triggers are arterial–venous oxygen difference and oxygen extraction ratio. Physiologic transfusion triggers reflect the patient-sensitive and time-sensitive balance between global oxygen delivery and consumption. The patient’s tolerance of anemia depends on this fine balance. The cut-off value for ScvO_2_/SvO_2_ (<55%), indicating a need for RBC transfusion, was derived from the published literature [19]. The cut-off value for blood lactate (>1.8 mmol/L) was based on the reference range from the local laboratory. Lactate was used in the past as a marker of an oxygen deficit, helping in the optimal timing of RBC transfusion [20]. Lactate was used as a physiologic transfusion trigger due to limited number of patients in whom ScvO_2_ could be determined. Central venous oxygen saturation can only be determined in patients with a central line, with the tip of a central line being located in the lower part of superior vena cava. To sum up, clinical scenarios in which RBC transfusion was deemed appropriate are presented in Table 1.

We classified as inappropriate all RBCs transfused outside the abovementioned clinical scenarios or RBCs transfused with no pre-transfusion Hb determination. At our institution, we support the one-unit transfusion policy [21]. The second unit of RBC transfused during a single-transfusion event was classified as inappropriate even if there were symptoms of anemia or positive laboratory markers of anaerobic metabolism prior to transfusion of the first unit, but no reassessment of Hb concentration, anemia signs/symptoms, or physiologic transfusion triggers was performed following the first RBC unit. Inappropriate RBC transfusions constituted RBC overuse. The incidence of RBC transfusions per 1000 patient-day was calculated as well as percentage of patients in whom at least a single RBC unit was transfused. We calculated the number of appropriately and inappropriately transfused RBCs overall and per hospital department.

Statistical analysis was performed using MedCalc version 18 statistical software (MedCalc Software, Ostend, Belgium). Continuous variables were presented as median and interquartile ranges (IQR) for variables with non-normal distribution. The type of distribution was verified with Shapiro–Wilk test. Categorical variables were expressed as numbers and percentages. Statistical significance was established by the chi-square or the Fischer’s exact test. Intergroup differences were evaluated by the Mann–Whitney test. The variables pre–post RBC transfusion were analyzed using the Wilcoxson test. *p* < 0.05 was considered statistically significant. Due to retrospective character of the study, the local ethics committee decided that the study did not require the committee’s approval (PCN/CBN/0022/KB/292/21).

## 3. Results

Out of 24,117 patients, 286 received at least a single RBC unit. The overall incidence of RBC transfusions at our institution was 10.2 per 1000 patient-days. The incidence of RBC transfusions and percentage of RBC-transfused patients in individual hospital departments are presented in Table 2.

The incidence of RBC transfusions was highest in the autoimmune and metabolic diseases department, the ICU, and the clinical pharmacology department. The percentage of RBC-transfused patients was also highest in these three locations.

Demographic and clinical characteristics of patients who were transfused with RBC are presented in Table 3.

The increase in Hb concentration following RBC transfusion was significant (*p* < 0.01), whereas drop in lactate concentration was not (*p* = 0.96). In 173 (20.9%) patients, pre-transfusion Hb was <70 g/L, and in 27 (3.3%) patients, lactate was >1.8 mmol/L; in 16 (1.9%), both criteria were met.

**Figure 1:**
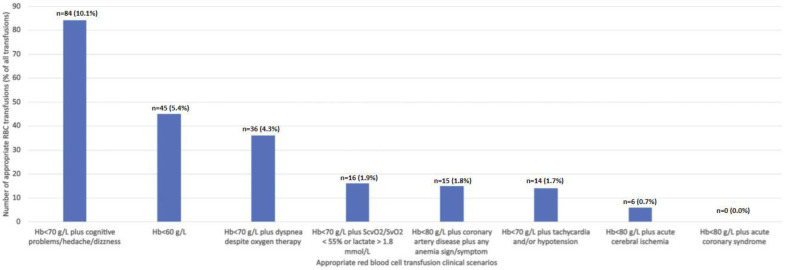
Number of appropriate RBC transfusions (percentage of all RBC transfusions) in various clinical scenarios classified as appropriate indications for RBC transfusion (Table 1). RBC, red blood cell.

During the 7-month period, there were 828 RBC units transfused to non-bleeding patients. There were 171 RBCs given as single-unit transfusions and 657 RBCs as multiple-unit transfusions. The numbers of RBCs that were transfused in clinical situations deemed appropriate indications for RBC transfusion are presented in Figure 1.

The most frequent clinical situations when RBC transfusion was classified as appropriate were: Hb < 70 g/L plus cognitive problems/headache/dizziness (10.1%), Hb < 60 g/L (5.4%), and Hb < 70 g/L plus dyspnea despite oxygen therapy (4.3%). Among patients with CAD in whom RBCs were transfused (n = 42), nine (21.4%) patients had cognitive symptoms, eight (19.0%) patients had dyspnea, and three (0.7%) patients had tachycardia and/or hypotension, while some patients had more than one sign/symptom. Among patients with and without CAD, the most frequent were neurological and respiratory sings/symptoms of anemia. There were 216 (26.1%) RBC units appropriately transfused and 612 (73.9%) RBC units that were transfused outside the abovementioned clinical scenarios. The most frequent causes of inappropriate RBC transfusion were: no Hb determination pre-RBC transfusion (n = 317) and among these RBC transfused as a second unit in a single-transfusion episode (n = 260), absence of anemia sings/symptoms (n = 179), and Hb concentration ≥80 g/L (n = 80). Incidence, number, and total volume of RBCs that were appropriately and inappropriately transfused is presented in Table 4.

Overall, 26.1% RBC transfusions were classified as appropriate and 73.9% as inappropriate. The incidence of appropriate and inappropriate RBC transfusions was 2.6 and 7.5 units per 1000 patient-days, respectively. Hospital departments with the highest percentage of appropriate RBC transfusions (>80%) were the neurology department (100%), ICU (83.7%) and stroke unit (83.3%). The percentage of appropriate RBC transfusions was <34% in all other hospital departments.

Comparison between patients who received appropriate and inappropriate RBC transfusions is presented in Table 5.

The patients who received appropriate and inappropriate RBC transfusions differed in Hb concentration pre–post RBC transfusion, whereas age, sex, CAD status, and lactate pre–post transfusion were not different.

## 4. Discussion

The overall percentage of RBC-transfused patients at our institution was 1.2%. This percentage was much lower than reported by Borkent-Raven et al. in 2011 (10.9%) [22] and 2010 (13%) [23]. This discrepancy might be due to different populations of patients and analyses performed in different time periods.

The aim of our study was to analyze the appropriateness of RBC transfusions in non-bleeding patients hospitalized at our institution. At our institution, 26.1% RBC transfusions were classified as appropriate. The highest percentage of appropriate RBC transfusions was in the neurology department, the ICU, and stroke unit (83.3–100%). This percentage was below 33% in all other hospital departments. In the study analyzing appropriateness of RBC transfusion in four emergency departments, 78.6% of RBC transfusions were considered appropriate. This high percentage of appropriate RBC transfusions was due to adopted criteria. Clinical scenarios in which RBC transfusion was considered appropriate were (based on the official consensus document from 2013): Hb < 70 g/L (acute anemia irrespective of sign/symptoms), Hb < 80 g/L (chronic anemia with symptoms of anemia or risk factors), Hb < 90 g/L (acute anemia with hemodynamic instability, CAD, heart failure, cerebrovascular disease, and myocardial infarction), and Hb ≥ 90 g/L (acute anemia with hemorrhage or symptoms of anemia) [24]. In our study, we did not consider Hb < 70 g/L without anemia symptoms or Hb < 90 g/L in patients with risk factors as appropriate indications for transfusion. To evaluate the appropriateness of RBC volume, the authors defined over-transfusion as post-transfusion Hb > 20 g/L above the appropriate transfusion trigger, and they showed an over-transfusion percentage of 45%. When this volume of over-transfusion was combined with inappropriate indication, 60% of RBC transfusion episodes and 41% of RBC units were found as unnecessarily transfused [24]. In the study by Shander et al., the panel of experts rated allogeneic RBC transfusions as inappropriate in 59.3% of nonbleeding clinical situations—all scenarios with Hb ≥ 100 g/L and 71.3% of scenarios with Hb 80–99 g/L. In 28.9% of clinical scenarios, RBC transfusion appropriateness was uncertain [25]. However, the authors did not take signs/symptoms of anemia into account when deciding on the appropriateness of RBC transfusion, unlike we did in our study.

The appropriateness of RBC transfusion in our study was based on the most recent AABB guidelines and some additional indications specified by the authors (Table 1). RBCs are transfused to improve the oxygen-carrying capacity of blood and delivery of oxygen to cells. Most clinical practice guidelines on RBC transfusion list specific Hb concentration thresholds as an indication for RBC transfusion. Clinical Practice Guidelines from AABB set the threshold at Hb ≤ 70 g/L in hospitalized hemodynamically stable patients and critically ill patients (strength of recommendation IB) [14]. The aforementioned guidelines set the Hb threshold at ≤80 g/L in patients undergoing orthopedic or cardiac surgery and in patients with coronary artery disease (strength of recommendation IB) although the Hb trigger ≤70 g/L and ≤80 g/L are most probably comparable in these patients [14]. Restrictive transfusion trigger (i.e., Hb 70–75 g/L) was shown to be safe compared to liberal transfusion trigger (i.e., 90–100 g/L) by randomized controlled trials in different populations of patients: the critically ill [26], cardiac surgery patients [27,28], orthopedic surgery patients with cardiac risk factors [29], septic patients [30], and those with malignant tumors [31]. All authors agreed with that RBC transfusion should be tailored to individual situations in accordance with the strategy of individualized therapy. The decision regarding RBC transfusion should be based on patient clinical condition and not only on Hb concentration [32,33]. Every patient has individualized adaptation mechanisms to anemia, so hemoglobin concentration cannot be the only marker of the need for RBC transfusion [34]. There are few exceptions in the context of the non-inferiority of restrictive RBC transfusion trigger. It is not known if, in myelodysplastic syndrome patients, restrictive liberal RBC transfusion trigger is more appropriate [35]. In patients with coronary artery disease (CAD), liberal RBC transfusion triggers should be taken into account [36]. Therefore, we classified RBC transfusions given to symptomatic patients with CAD with Hb < 80g/L as appropriate. Guidelines from AABB also suggest considering clinical context (clinical signs or symptoms of anemia) when deciding on RBC transfusion in non-bleeding patients [13,14]. Although transfusion guidelines are meant as a support tool, they should not replace the clinical judgement of clinicians making decisions on RBC transfusions. There may be situations when patients do not tolerate anemia with Hb concentrations above the restrictive trigger (e.g., myelodysplastic syndrome patients); on the other hand, young, otherwise healthy, chronically anemic patients may tolerate Hb < 6 g/dL well and not require RBC transfusion. The imperative here is to look for signs and symptoms of anemia. The supporting role in the decision regarding RBC transfusions may have physiological transfusion triggers. The most widely available is lactate concentration. Nevertheless, clinicians should be aware of multiple factors influencing lactate concentration in sepsis patients [37]. Moreover, RBC transfusion may not produce a clinically sound drop in lactate concentration, making reassessment following RBC transfusion problematic [38]. In our study, the most frequently reported symptoms of anemia were neurological symptoms. This result is not unusual, as high oxygen consumption makes the brain particularly sensitive to anemia. Some studies even suggest that anemia is strongly associated with overall cognitive impairment and may be a risk factor for dementia (1.39, 95% CI 1.23–1.56, *p* < 0.001), Alzheimer’s disease (1.59, 95% CI 1.18–2.13, *p* = 0.002), and mild cognitive impairment (1.36, 95% CI, 1.04–1.78; *p* = 0.02) [39,40]. On the other hand, Valladao et al. did not notice the association between worse cognitive function or mental disorders and anemia [41]. To reduce unnecessary transfusions, Edwards et al. suggested examining Hb concentration after single RBC transfusion [42]. In our study, multiple-unit RBC transfusions were also classified as inappropriate. Multiple-unit RBC transfusion was also the main cause of inappropriate transfusion in our study. Discharge Hb concentration may be representative and effective retrospective indicator of appropriateness of RBC transfusion [42]. Data on discharge Hb concentration were not retrieved in our study.

Although several guidelines have been proposed to reduce the RBC overuse, our study showed that the unjustified use of RBC persists. Some studies suggest that the decision regarding RBC transfusion may be based on beliefs and fixed behaviors [43,44]. Attempts at the reduction of inappropriate RBC transfusions is especially valid in the context of the low number of blood donors and the ever-increasing demand for blood [45]. Implementation of standardized RBC guidelines, medical staff education, and prospective transfusion-order screening may lead to the reduction of inappropriate RBCs transfusions [46]. Therefore, there is a definite need to educate physicians on appropriate indications for RBC transfusion. The educational interventions may have different results. The traditional approach to educate on appropriateness of RBC transfusions is through direct training campaigns; however, their effectiveness may be limited [47]. Introduction of clinical decision-support (CDS) electronic alerts is potentially more effective. Goodnough et al. showed that transfusions with pre-transfusion Hb > 80 g/L decreased from 60% to 30% following the introduction of CDS [48]. Tavares at al. showed that engagement of physicians may result in a 33% decrease in total number of RBC transfusions [49]. A significant reduction number of inappropriate RBC transfusion—from 25% to 15% of total RBC transfused in the medical-surgical ICU—was noticed after implementation of a transfusion bundle [50]. Introduction of patient blood management (PBM) may lead to more appropriate RBC transfusions in medical and surgical departments [51,52]. Ordering physicians may override the alerts on proper RBC transfusion indicators, and the main reason for this behavior is “protocolized behaviors on specialty services” [53]. Conflicting practices in various clinical specialties, potential costs, and differences in skills and knowledge of co-workers are indicated as the most important impediments to RBC transfusions based on evidence [54].

The last step in the efforts to minimize the incidence of inappropriate RBC transfusions may be the patient’s informed consent for the procedure. Such consent or lack of it should be the result of thorough discussion with the patient about benefits, risks, alternatives, and consequences of refusal of RBC transfusion. In our study, we did not look into the informed consent process specifically. Although at our institution, the consent form for RBC transfusion includes statement on alternative management, we do not know how many patients were offered alternative treatment options.

Our study is not without limitations. Due to the retrospective character of the study, we did not have access to some important clinical information that was not documented in the patients’ case notes. Although we thoroughly analyzed all daily entries of the patients on the day of RBC transfusion, there is a possibility that signs or symptoms of anemia were not properly documented by ordering physicians, and some RBC transfusion episodes could be wrongly evaluated as inappropriate. The presented indications for appropriate RBC transfusions may be judged by some authors as debatable. Some patients (e.g., myelodysplastic syndrome patients) may require RBC transfusion to increase Hb concentration above 80 g/L, which according to our approach was evaluated as inappropriate.

## 5. Conclusions

Although the incidence of RBC transfusions in non-bleeding inpatients in our study was generally low, the majority of RBC transfusions was performed outside recommended indications. RBC transfusions were rated as inappropriate mainly due to multiple-unit transfusion episodes, absence of anemia signs and/or symptoms before transfusion, and adoption of liberal transfusion triggers. There is still need to educate physicians on appropriate indications for RBC transfusion.

## Figures and Tables

**Table 1:** Appropriate red blood cell transfusion clinical scenarios.

Clinical Scenarios
Hb ^1^ < 60 g/L;
Hb < 70 g/L plus tachycardia and/or hypotension despite normal blood volume;
Hb < 70 g/L plus dyspnea and/or tachypnea despite oxygen therapy (aimed at SpO_2_ ^2^ 100%);
Hb < 70 g/L plus problems with concentration/attention and/or headache and/or dizziness;
Hb < 70 g/L plus Scv_2_O ^3^/SvO_2_ ^4^ < 55% and/or lactate > 1.8 mmol/L;
Hb < 80 g/L plus coronary artery disease plus any sign/symptom of anemia;
Hb < 80 g/L plus acute coronary syndrome;
Hb < 80 g/L plus acute cerebral ischemia.

^1^ Hemoglobin; ^2^ peripheral oxygen saturation; ^3^ central venous oxygen saturation; ^4^ mixed venous oxygen saturation.

**Table 2:** Incidence of red blood cell transfusions and percentage of transfused patients.

Hospital Department	Incidence of RBC ^1^ Transfusions (Unit/1000 Patient-Days)	RBC-Transfused Patients (%)
Autoimmune and Metabolic Diseases	41.8	8.8
Intensive Care Unit	27.6	8.9
Clinical Pharmacology	25.6	7.4
Gastroenterology and Hepatology	18.7	2.9
Gastrointestinal Surgery	17.1	3.8
Gynecology and Obstetrics	14.5	1.9
Oncologic Surgery	7.7	0.7
Radiotherapy	5.2	0.8
Medical Oncology (2 units)	4.6	0.3
Neurosurgery	3.8	0.9
Stroke Unit	3.6	1.6
Endocrinology	1.6	0.2
Allergology and Immunology	1.1	0.1
Neurology	0.8	0.2
Neurologic Rehabilitation	0.5	0.9
Ophthalmology	0.2	<0.1
All departments	10.2	1.2

^1^ Red blood cell.

**Table 3:** Characteristics of patients transfused with red blood cell units.

Patient Characteristic	Value
Age, median, IQR ^1^ (years)	65 (47–75)
Sex, male/female (n; %)	120 (42.0)/166 (58.0)
Coronary artery disease (n; %)	42 (14.7)
Hb ^2^ concentration pre RBC ^3^ transfusion, median, IQR (g/L)	75 (65–80)
Hb concentration post RBC transfusion, median, IQR (g/L)	85 (77–95)
Patients with pre RBC transfusion Hb < 70 g/L, number, %	173 (20.9)
Lactate concentration pre RBC transfusion, median, IQR (mmol/L)	2.0 (1.5–2.6)
Lactate concentration post RBC transfusion, median, IQR (mmol/L)	1.8 (1.4–2.4)
Patients with pre RBC transfusion lactate > 1.8 mmol/L, number,%	27 (3.3)

^1^ Interquartile range; ^2^ hemoglobin; ^3^ red blood cell.

**Table 4:** Incidence, number, and volume of appropriate and inappropriate red blood cell transfusions per hospital department.

Hospital Department	Appropriate RBC Transfusions		Inappropriate RBC Transfusions	
	Incidence (n/1000 Patient-Days)	RBC * (%/n)	Incidence (n/1000 Patient-Days)	RBC (%/n)
Neurology	0.8	100/3	0.0	0/0
Intensive Care Unit	23.1	83.7/41	4.5	16.3/8
Stroke Unit	2.9	83.3/10	0.6	16.7/2
Endocrinology	0.5	33.3/2	1.1	66.7/4
Clinical Pharmacology	8.1	31.6/30	17.5	68.4/65
Gastrointestinal Surgery	4.7	27.5/28	12.4	72.5/74
Gastroenterology and Hepatology	4.4	23.6/39	14.3	76.4/126
Medical Oncology (2 units)	1.1	22.9/8	3.6	77.1/27
Neurosurgery	0.8	20.0/4	3.0	80.0/16
Autoimmune and Metabolic Diseases	8.2	19.5/34	33.7	80.5/140
Gynecology and Obstetrics	1.7	11.5/15	12.8	88.5/115
Oncologic Surgery	0.9	11.1/2	6.8	88.9/16
Neurologic Rehabilitation	0.0	0/0	0.5	100/2
Ophthalmology	0.0	0/0	0.2	100/2
Allergology and Immunology	0.0	0/0	1.1	100/2
Radiotherapy	0.0	0/0	5.2	100/13
All departments	2.6	26.1/216	7.5	73.9/612

* Red blood cell.

**Table 5:** Characteristics of recipients of appropriate and inappropriate RBC transfusions.

Characteristic	Appropriate RBC ^1^ Transfusion	Inappropriate RBC Transfusion	*p*
Age, median, IQR ^2^ (years)	66.5 (50–78)	64.5 (45.5–74.0)	0.50
Sex, male/female (n)	31/35	89/131	0.35
Coronary artery disease (n; %)	14 (20.6)	28 (79.4)	0.09
Hb ^3^ concentration pre RBC, median, IQR (g/L)	**64 (58–68)**	**75 (70–80)**	**<0.01**
Hb concentration post RBC, median, IQR (g/L)	**80 (73–90)**	**95 (87–10)**	**<0.01**
Lactate concentration pre RBC, median, IQR (mmol/L)	2.0 (1.5–2.7)	1.7 (1.2–2.2)	0.28
Lactate concentration post RBC, median, IQR (mmol/L)	1.9 (1.4–2.5)	2.0 (1.3–2.3)	0.73

^1^ Red blood cell; ^2^ interquartile range; ^3^ hemoglobin. In bold: statistically significant values.

## Data Availability

The data presented in this study are available on request from the corresponding author.
